# Integrated Analysis of Transcriptome and Metabolome Reveals Metabolite Biosynthesis in Pigmented Potatoes

**DOI:** 10.3390/ijms27062881

**Published:** 2026-03-23

**Authors:** Gongkai Chen, Fanglei Hong, Lingli Wang, Yichuan Zhang, Hong Wang, Shuangshuang Xin, Hongshuang Yang, Kang Ning, Yong’an Liu

**Affiliations:** 1 Wenzhou Institute of Agricultural Sciences, Wenzhou 325006, China; 2 Key Laboratory of Crop Breeding South Zhejiang, Wenzhou 325006, China; 3 College of Life Sciences, China Jiliang University, Hangzhou 314423, China

**Keywords:** potato, secondary metabolites, flavonoids, RNA-seq

## Abstract

Potato (*Solanum tuberosum* L.), the fourth most important food crop worldwide, serves as a multi-purpose resource for food, feed and industrial raw materials, and plays a pivotal role in safeguarding food security, diversifying dietary structure and boosting the development of agricultural economy. With increasing consumer demand for nutritional quality, elucidating the regulatory mechanisms of potato quality traits has become a research priority. In this study, three potato cultivars with distinct coloration were employed as materials. Metabolomic profiling identified a total of 1128 metabolites, and revealed that pigmented potato cultivars accumulated higher levels of flavonoids and linoleic acid derivatives compared with the white-fleshed cultivar. Transcriptomic analysis uncovered numerous differentially expressed genes (DEGs) among the three cultivars; notably, DEGs in pigmented cultivars were significantly enriched in pathways related to terpenoid backbone biosynthesis, flavonoid biosynthesis, linoleic acid metabolism, and starch and sucrose metabolism. Integrated multi-omics analysis revealed that the high expression of structural genes in the flavonoid biosynthesis pathway is strongly associated with flavonoid accumulation in pigmented potatoes, suggesting that transcriptional upregulation of these genes may be a key driver of flavonoid biosynthesis. Furthermore, several MYB and WD40 family transcription factors were identified as potential regulators of flavonoid and anthocyanin biosynthesis in potato. Collectively, our study provides insight into the regulatory mechanisms underlying the biosynthesis of secondary metabolites in potato by combining transcriptomic and metabolomic approaches, and the findings provide a valuable theoretical basis for the genetic improvement of potato nutritional quality in future breeding programs.

## 1. Introduction

Potato, an annual herbaceous species of the genus *Solanum* (*Solanaceae*), is the world’s third most important staple food crop following rice and wheat, with a global annual production of approximately 380 million metric tons. Characterized by robust environmental adaptability, this crop can be successfully cultivated in marginal croplands such as high-altitude, high-latitude and arid regions, thereby emerging as a pivotal species for expanding arable land resources and alleviating cultivation land constraints [1]. In terms of production efficiency, potato yields 1.3-fold more calories and 2.2-fold more protein per unit area compared with wheat; such superior productivity endows it with prominent strategic reserve value for safeguarding global food security under the backdrop of escalating food demand and climate change [2,3]. Besides its important role in food security, potato also boasts excellent nutritional and economic value. It is rich in carbohydrates, vitamin C, potassium, and dietary fiber, while having an extremely low fat content, thus has been recognized by the World Health Organization (WHO) as an “economical and nutritionally balanced food” [4].

Plant secondary metabolites are small-molecule organic compounds that, while traditionally distinguished from primary metabolites essential for growth and development, play crucial roles in plant survival and fitness. These compounds mediate ecological interactions including defense against pathogens and herbivores, signaling for symbiosis establishment, and adaptation to abiotic stresses, thereby directly impacting growth and reproductive success under natural conditions [5]. Furthermore, many secondary metabolites interact with and modulate primary metabolic pathways, forming an integrated metabolic network essential for plant-environment adaptation. Mediating the interactions between plants and their biotic and abiotic surroundings, these metabolites constitute the core survival strategy of plants [6]. While humans ingest plant primary metabolites to meet basic physiological needs, plant secondary metabolites also exert significant physiological regulatory and health-promoting effects on human health. For instance, typical secondary metabolites including anthocyanins, flavonoids and terpenoids, all supplement human nutrition, regulate metabolism, and perform vital functions such as antioxidation and anti-inflammation [7,8,9]. With improving of people’s living standards, the demand for potato has shifted from mere satiety to higher nutritional quality, and new potato cultivars rich in functional nutrient factors have gradually gained market attention.

Flavonoids and anthocyanins are representative polyphenolic secondary metabolites widely distributed in angiosperms [10]. Though not essential for plant growth and development, they are key adaptive substances evolved in plants and play a synergistic core role in stress resistance, pollinator attraction and other processes [6]. Flavonoids can inhibit pathogen infection and act as copigments to enhance the stability and physiological activity of anthocyanins; together, they endow plants with bright colors. Meanwhile, they can scavenge reactive oxygen species and alleviate membrane lipid peroxidation damage through antioxidant effects, serving as an important material basis for plants to resist biotic and abiotic stresses. Terpenoids are the core group with the richest variety and most diverse functions among plant secondary metabolites, synthesized with isoprene as the basic skeleton and widely distributed in various organs and tissues of plants. Terpenoids are not essential for plant growth and development, but they are key adaptive components formed during long-term plant evolution, running through the entire process of plant growth regulation, environmental adaptation, interspecific interaction and population reproduction [11]. Anthocyanin biosynthesis is a crucial branch of the flavonoid metabolic pathway in plants, which is catalyzed by a series of enzymes encoded by structural genes, including phenylalanine ammonia-lyase (PAL), chalcone synthase (CHS), chalcone isomerase (CHI), flavanone 3-hydroxylase (F3H), dihydroflavonol 4-reductase (DFR), anthocyanidin synthase (ANS) and UDP-glycosyltransferase (UFGT) [12]. The transcription of these structural genes is mainly regulated by the highly conserved MYB–bHLH–WD40 (MBW) transcription factor complexes, in which MYB transcription factors determine the specificity of target gene recognition, bHLH transcription factors enhance the binding activity to promoters, and WD40 proteins act as scaffold molecules to stabilize the whole complex; the MBW complex synergistically activates the expression of late biosynthetic genes, thereby promoting anthocyanin accumulation, coloring, and adaptation to environmental stresses such as light and low temperature [13]. The tuber color of potato, such as purple, red, yellow, or white, is an important trait concerned by consumers. Among them, purple and red potato cultivars are rich in flavonoids and anthocyanins, which endow them with unique nutritional and health-care values such as antioxidant and anti-inflammatory properties.

In this study, we investigated the transcriptomic and metabolomic differences in tubers among three potato cultivars (pigmented and white-fleshed cultivars). We identified a large number of differentially expressed genes and metabolites among different cultivars, and the tubers of colored cultivars were richer in terpenoids and flavonoids. Several transcription factors potentially involved in the regulation of anthocyanin biosynthesis in potato were screened through integrated analysis. These findings provide important theoretical and practical value for improving the nutritional value of potato, expanding its industrial applications, and meeting the diverse demands of the market.

## 2. Results and Discussion

### 2.1. Metabolomics Profiling of Three Potato Cultivars (AG594, DJ247 and DG26)

**Figure 1:**
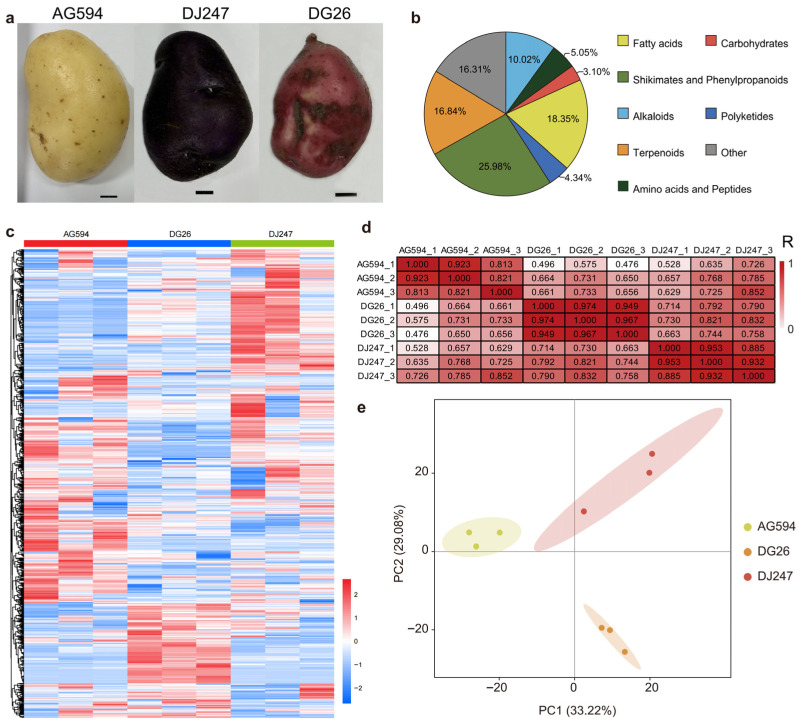
Metabolomics Profiling of three potato cultivars. (**a**) Image of three potato cultivars. (**b**) Classification statistics of identified metabolites. Percentages may not total 100 due to rounding. (**c**) Correlation heatmap of biological replicates of samples. (**d**) Metabolite heatmap. (**e**) PCA analysis of samples.

The potato tuber is a subterranean stem modification of the potato plant, formed by the swelling of the tip of a stolon. It serves as both a storage organ for nutrients and a vegetative propagation organ. Three potato cultivars with visibly distinct tuber phenotypes were selected for further analysis (Figure 1a). By using Untargeted LC-MS/MS analysis, 1128 metabolites (VIP > 1, *p* < 0.05) were detected, of which 25.98% were annotated to shikimates and phenylpropanoids, 16.84% to terpenoids and 18.35% to fatty-acid-related compounds (Figure 1b,c, Table S1). Shikimic acid and phenylpropanoid metabolites are known to be involved in plant defense responses and adaptation to abiotic stresses based on literature evidence [14,15]. Phenylpropanoids (e.g., chlorogenic acid, phenolic acids) exert a significant inhibitory effect on soil-borne pathogenic fungi, which indicates the continuous role of potato tubers in resisting pathogenic infection [16,17]. The high accumulation of these metabolites in all three cultivars suggests their potential role in tuber defense, though direct experimental validation of disease resistance was not performed in this study. Flavonoids are potent antioxidants that can scavenge reactive oxygen species (ROS) generated during respiration, thus protecting starch and proteins from oxidative damage [18]. Lignans such as neolignans may help strengthen tuber periderm structure, though their specific role in these cultivars requires further functional validation. Metabolomic analysis reveals that flavonoids such as naringin, kaempferol, and quercetin, which share common biological functions including antioxidant, anti-inflammatory and cardiovascular protection, show significant differential accumulation among different cultivars. Therefore, differences in the content of these substances are a key factor driving the differentiation of color and quality among the three cultivars. Lignans such as neolignans helps strengthen the structure of tuber periderm and vascular bundles, physically preventing the invasion of pathogens [19]. Terpenoids play a central role in disease resistance, insect resistance, signal transduction, and ecological interactions. For example, the sesquiterpene alcohol-type plant antibiotic rishitinol is a key chemical barrier for potatoes to resist the late blight pathogen [20]. The level of sugars in potato tuber is an important factor affecting quality in potatoes, especially colour of processed products [21]. Many monosaccharides (glucose, fructose, galactose) and disaccharides (sucrose, trehalose, maltose) were detected in the three cultivars, with notable differences in accumulation patterns. For instance, sucrose and trehalose showed significantly higher levels in DG26 compared to AG594, which may contribute to the sweetness and processing quality differences among cultivars.

The metabolomics data showed that among the top 10 metabolites with the highest content in each of the three cultivars individually, linolenic acid and its homologues (including alpha-linolenic acid and gamma-linolenic acid) were consistently detected across all cultivars. This indicates that linolenic acid derivatives are among the most abundant metabolites regardless of flesh color phenotype. Linolenic acid is an important polyunsaturated fatty acid in potato tubers that serves as a core component of membrane phospholipids to maintain structural and functional homeostasis of the membrane system, acts as a key precursor for jasmonic acid synthesis to mediate biotic/abiotic stress defense, scavenges reactive oxygen species to delay cellular oxidative senescence, and regulates carbon and nitrogen metabolism and starch accumulation to enhance nutritional quality and extend postharvest storage period, thereby constituting a crucial lipid substance for ensuring the physiological functions, commercial value, and stress resistance of potato tubers [22]. The metabolites such as methyl pentadecanoate, 3-epioleanolic acid, gamma-linolenic acid, LPE(18:1(9Z)/0:0), isocaproic acid, 1-(1H-indol-3-yl) ethanone, alpha-linolenic acid, (Z)-2-hydroxyoctadec-9-enoic acid and myristoleic acid exhibit the largest differential abundance between 3 cultivars. The tubers of the three potato cultivars exhibit significant differences in color.

Pearson correlation coefficients among biological replicates ranged from 0.81 to 0.97 (Figure 1d). Principal component analysis (PCA) revealed clear separation of AG594, DG26 and DJ247 along PC1 and PC2, which together explained 33.22% and 29.08% of the total variance, respectively (Figure 1e). These result indicated that these two principal components can represent the variability of the original data well.

### 2.2. Metabolites Profile of Three Cultivars

**Figure 2:**
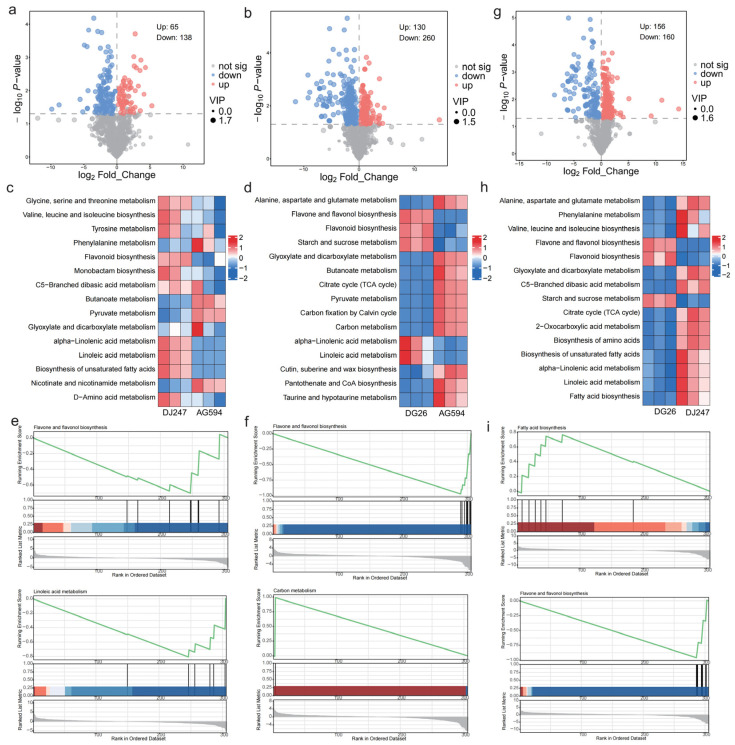
Differentially accumulated metabolites (DAMs) between cultivars. Volcano plot of DG26 vs. AG594 (**a**), DJ247 vs. AG594 (**b**) and DG26 vs. DJ247 (**g**). Heatmap of KEGG enriched pathways in DG26 vs. AG594 (**c**), DJ247 vs. AG594 (**d**) and DG26 vs. DJ247 (**h**). GSEA of item that enriched in different cultivars (**e**–**i**). The green line shows the Running Enrichment Score (RES) of the pathway. Black vertical bars mark the positions of pathway genes in the ranked gene list. The gray curve displays the gene-level ranked metric (e.g., log_2_FC) indicating phenotype association.

Then we compared the differentially accumulated metabolites (DAMs) between the these cultivars. First we compared the pigmented cultivars with white-fleshed cultivar (DG26 vs. AG594 and DJ247 vs. AG594). Compared with white-fleshed cultivar (AG594), DG26 and DJ247 showed 130 and 65 metabolites highly accumulated, respectively (Figure 2a,b, Tables S2 and S3). KEGG showed that these DAMs are enriched in Flavone and flavonol biosynthesis, Linoleic acid metabolism and alpha−Linolenic acid metabolism pathways (Figure 2c,d). Flavonols (such as rhoifolin, rutin, and nicotiflorin) share upstream biosynthetic pathways with anthocyanins [23]. Metabolomic analysis showed that several flavonoids such as rhoifolin, Rutin, Kaempferol 3-O-sophoroside and Kaempferol-3-O-galactoside were highly accumulated in the pigmented cultivars, suggesting an overall increased metabolic flux of the flavonoid pathway, which elevated the contents of both flavonols and anthocyanins. Linoleic acid metabolism related metabolites like Linoleic acid, gamma-Linolenic acid, 9-HPODE, 13(S)-HODE, 13(S)-HpODE were also highly accumulated in the pigmented cultivars. KEGG also showed that Starch and sucrose metabolism were highly accumulated in the DG26 and DJ247 compared with AG594 respectively. For example Carbohydrates like Sucrose, trehalose and Isomaltose were highly accumulated in DG26 compared with AG594. While, Glyoxylate and dicarboxylate metabolism, Butanoate metabolism and Pyruvate metabolism related metabolites such as Pyruvate, glutamate and maleic acid were enriched in AG594. GSEA (Gene Set Enrichment Analysis) showed that flavone and flavonol biosynthesis related metabolites were highly accumulated in the pigmented cultivars (DJ247 and DG26), while linoleic acid metabolism and carbon metabolism related metabolites were highly accumulated in AG594. Therefore, the color difference between the two pigmented cultivars is mainly attributed to the enrichment of flavonoid pathway-related metabolites (Figure 2e,f).

We also compared the pigmented cultivars (DG26 vs. DJ247), totally 316 metabolites were detected between two pigmented cultivars (156 highly accumulated in DG26 and 160 highly accumulated in DJ247) (Figure 2g, Table S4). KEGG analysis and GSEA showed that Flavone and flavonol biosynthesis, Flavonoid biosynthesis pathway related metabolites were highly accumulated in DG26 (Figure 2h,i). For example, Cyanidin 3-[6-(4-glucosylcoumaryl) sophoroside] 5-glucoside, Flavone base + 3O, C-Hex-CoumaroylHexwere, Kaempferol 3-neohesperidoside and Kaempferol 3-O-gentiobioside and Rutin were highly accumulated in DG26. While 2−Oxocarboxylic acid metabolism and biosynthesis of unsaturated fatty acid related metabolites were enriched in DJ247.

### 2.3. Sequencing of the Potato Transcriptome Using RNA-Seq

**Figure 3:**
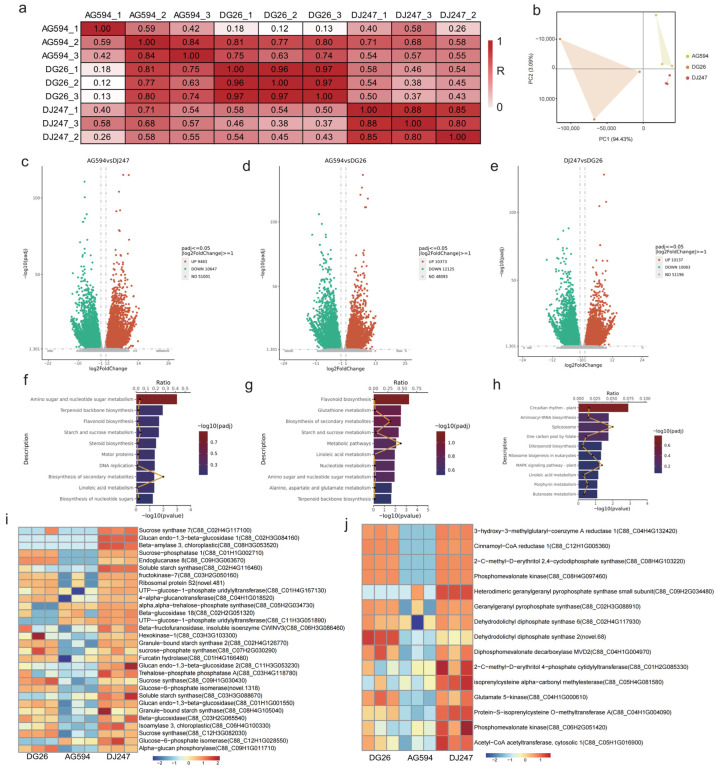
Transcriptional Profiling between three cultivars. (**a**) Co-relationship between the three cultivars. (**b**) PCA of three cultivars. DEGs number identified in AG594_DJ247 (**c**), AG594_DG26 (**d**) and DJ247_DG26 (**e**). Bar chart of KEGG pathway enrichment for DEGs across AG594_DJ247 (**f**), AG594_DG26 (**g**) and DJ247_DG26 (**h**). Heatmap of genes involved in starch and sucrose metabolism (**i**) and terpenoid backbone biosynthesis pathways (**j**). Z-score value of normalized FPKM of gene was used to draw heatmap.

To further explore the mechanism of the biosynthesis of secondary metabolites, we performed comparative transcriptome analysis among three cultivars. Approximately 38.5–44.3 million raw reads were generated from 9 samples (three replicates of each cultivar). After quality control, the percentage of bases with a Phred quality score ≥ 30 (Q30) exceeded 97%, indicating high-quality sequencing data. Over 90% of the clean reads were successfully mapped to the reference genome (Tables S5 and S6). PCA showed that three cultivars separated from each other, but replicates were clustered together, suggesting that our transcriptome data is reliable (Figure 3a,b).

First, we compared the Differentially Expressed Genes (DEGs) between these cultivars. AG594 (white-fleshed cultivar) was used as the reference baseline for comparisons with pigmented cultivars. In the DJ247 vs. AG594 comparison, 10,647 genes were upregulated and 9483 genes were downregulated in DJ247 relative to AG594. In the DG26 vs. AG594 comparison, 10,373 genes were upregulated and 12,125 genes were downregulated in DG26 relative to AG594. For the direct comparison between pigmented cultivars (DJ247 vs. DG26), 10,137 genes were highly expressed in DJ247, while 10,083 genes were highly expressed in DG26 (Figure 3c–e, Tables S7–S9). The large number of differentially expressed genes reflects the substantial genetic and metabolic divergence between pigmented and white-fleshed potato cultivars. These differences may stem from their distinct breeding lineages and selection histories. In addition, the autotetraploid nature of potato, which allows for differential expression among homoeologous gene copies, may also contribute to the extensive transcriptomic variations observed in this study.

Then we annotated the DEGs in the GO and KEGG databases. Compared with white-fleshed cultivar AG594, genes that highly expressed in the pigmented cultivars (DJ247 and DG26) were related with pathways like terpenoid backbone biosynthesis, flavonoid biosynthesis, starch and sucrose metabolism and linoleic acid metabolism (Figure 3f–h). Differential genes expression in pathways such as diterpenoid biosynthesis, linoleic acid metabolism, porphyrin metabolism, and butyrate metabolism may lead to differences in secondary metabolites between DJ247 and DG26. It is worth noting that the differentially expressed genes between the two cultivars also involve pathways such as the plant circadian rhythm, spliceosome, and plant MAPK signaling pathway. These pathways have been reported to participate in plant growth regulation, stress responses, and metabolic processes. For example, the plant circadian clock is a core regulatory hub that influences growth rhythms, stress responses, and substance accumulation by modulating the spatiotemporal expression of downstream genes; The spliceosome participates in post-transcriptional splicing regulation, directly affecting mRNA maturation, localization, and translation efficiency. It serves as a crucial component of epigenetic and post-transcriptional regulation, mediating expression diversity of genes associated with the circadian clock and signaling pathways; the plant MAPK signaling pathway is a core transduction route for cellular responses to exogenous/endogenous signals (e.g., environmental stress, hormonal signals, circadian clock signals), capable of converting extracellular signals into intracellular gene expression changes while also reversibly regulating the expression of circadian clock- and spliceosome-related genes. Thus, these pathways may also be involved in the metabolic processes of potato. However, the synergistic regulatory network warrants further experimental investigation.

Genetic differentiation within this network, by regulating downstream functional gene expression, ultimately leads to significant phenotypic differences between the two cultivars in growth rhythms, environmental responses, and other traits.

Several starch and sucrose metabolism pathway genes, including sucrose synthase (C88_C02H4G117100, C88_C09H1G030430, and C88_C12H3G082030), Soluble starch synthase (C88_C02H4G116460 and C88_C03H3G088670), and Sucrose-phosphate synthase (C88_C07H2G030290), were highly expressed in DJ247 and DG26 (Figure 3i). These starch and sucrose metabolism genes (sucrose synthases, soluble starch synthases, and sucrose-phosphate synthase) may be responsible for the altered carbohydrate profiles in DJ247 and DG26 cultivars compared to AG594, which could indirectly influence flavonoid accumulation through carbon partitioning. Similarly, genes involved in terpenoid backbone biosynthesis, such as Cinnamoyl-CoA reductase 1 (C88_C12H1G005360), Phosphomevalonate kinase (C88_C08H4G097460), Geranylgeranyl pyrophosphate synthase (C88_C02H3G088910), Dehydrodolichyl diphosphate synthase (C88_C02H4G117930), Diphosphomevalonate decarboxylase (C88_C04H1G004970), Glutamate 5-kinase (C88_C04H1G000610), Phosphomevalonate kinase (C88_C06H2G051420), and Acetyl-CoA acetyltransferase (C88_C05H1G016900), may be key genes responsible for the differential accumulation of terpenoid compounds in potato (Figure 3j).

### 2.4. Integration of Metabolome and Transcriptome Data

**Figure 4:**
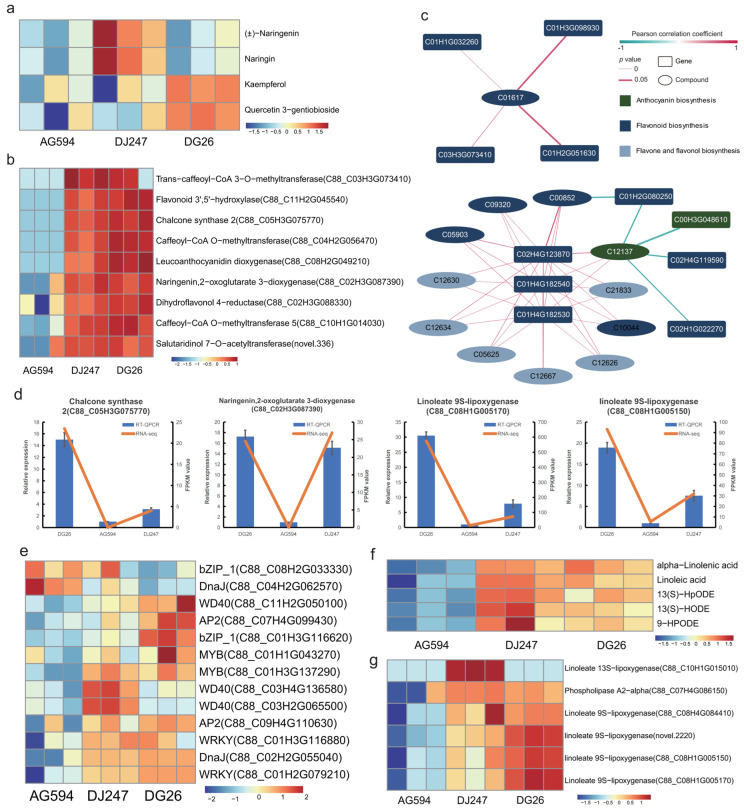
Integration of metabolome and transcriptome. Heatmap of flavonoid biosynthesis pathway compounds (**a**) and gene expression (**b**). (**c**) Network of genes and compound of Anthocyanin biosynthesis and Flavonoid biosynthesis pathways. Red/green edges indicate significant positive/negative Pearson correlations (*p* ≤ 0.05), with line thickness representing p-value (thicker = more significant). (**d**) RT-qPCR analysis and RNA-seq data of 4 genes. The RT-qPCR data are shown in the blue column, while the RNA-seq data are represented by the orange line. (**e**) heatmap of transcription factors. Z-score value of normalized FPKM of gene was used to draw heatmap. Heatmap of linoleic acid metabolism compounds (**f**) and gene expression (**g**).

In order to conduct a more comprehensive and integrated analysis of the differences between three cultivars, we performed integrated transcriptome-metabolism analyses. Both metabolome and transcriptome data revealed significant differences in linoleic acid- and flavonoid-related metabolites and biosynthetic pathway genes among the three cultivars. We focused on these two classes of compounds and their biosynthetic genes. Key flavonoid and anthocyanin biosynthesis pathway compounds, such as (±)-Naringenin, Kaempferol, Naringin, and Quercetin 3-gentiobioside, accumulated to higher levels in DG26 and DJ247 than in AG594 (Figure 4a). Several flavonoid biosynthesis pathway gene like Trans-caffeoyl-CoA 3-O-methyltransferase (C88_C03H3G073410), Flavonoid 3′,5′-hydroxylase (C88_C11H2G045540), Chalcone synthase 2 (C88_C05H3G075770), Caffeoyl-CoA O-methyltransferase (C88_C04H2G056470), Leucoanthocyanidin dioxygenase (C88_C08H2G049210), Naringenin,2-oxoglutarate 3-dioxygenase (C88_C02H3G087390), Dihydroflavonol 4-reductase (C88_C02H3G088330), Caffeoyl-CoA O-methyltransferase 5 (C88_C10H1G014030), Salutaridinol 7-O-acetyltransferase (novel.336) were highly expressed in DG26 and DJ247, but have a low expression in AG594 (Figure 4b). It can be seen that these anthocyanin/flavonoid biosynthesis pathway genes form an interaction network to participate in the synthesis of flavonoids/anthocyanins (Figure 4c). Several flavonoid biosynthesis pathway genes (Chalcone synthase 2, Naringenin,2-oxoglutarate 3-dioxygenase)were chosen to perform RT-qPCR. The result showed that two selected genes showed similar expression pattern in RNA-seq and RT-qPCR, which indicating that our RNA-seq were reliable (Figure 4d). Studies showed that MYB and WD40 types of transcription factors were involved in flavonoid/anthocyanin biosynthesis, by binding to the promoter of key pathway genes [24]. Several MYB and WD40 family transcription factors were identified as putative candidate regulators of flavonoid and anthocyanin biosynthesis in potato based on their co-expression patterns with structural pathway genes (Figure 4e). However, functional validation through approaches such as overexpression, RNAi, transient promoter-binding assays, or mutant analysis is required to establish causative regulatory relationships.

Linoleic acid metabolism pathways related metabolites such as alpha-Linolenic acid, Linoleic acid, 13(S)-HODE, 13(S)-HpODE, 9-HPODE were highly accumulated in DG26 and DJ247. And Linoleic acid metabolism pathways related genes such as Linoleate 13S-lipoxygenase (C88_C10H1G015010), Phospholipase A2-alpha (C88_C07H4G086150), Linoleate 9S-lipoxygenase(C88_C08H4G084410, novel.2220, C88_C08H1G005150 and C88_C08H1G005170) were highly expressed in DG26 and DJ247 (Figure 4f,g). Transcription factors were selected based on their expression pattern (Figure 4e). Several TFs (C88_C09H4G110630, C88_C01H3G116880, C88_C02H2G055040 and C88_C01H2G079210) were highly expressed in DG26 and DJ247, while TFs (C88_C08H2G033330 and C88_C04H2G062570) were specificly expressed in AG594. These TFs may be regulatory factors involved in the regulation of specific secondary metabolites in different potato cultivars. However, these candidate transcription factors were only screened and predicted based on their expression patterns in the present study. Further experimental evidence, such as yeast one-hybrid assays, dual-luciferase reporter assays, and transgenic verification, is still lacking to confirm their direct regulatory relationships with target genes and their actual functions in the metabolism of specific secondary metabolites.

## 3. Materials and Methods

### 3.1. Plant Materials and Sampling

This study selected three potato cultivars with significant differences in tuber color as experimental materials, namely the conventional yellow cultivar AG594, the purple cultivar DJ247, and the red cultivar DG26. Field experiments were conducted at the Wenzhou Institute of Agricultural Sciences experimental farm (28°03′ N, 120°39′ E, elevation 15 m), Zhejiang Province, China, during the 2023 growing season (March–July). Plants were grown under natural sunlight conditions with standard agricultural practices including regular irrigation, fertilization, and pest management following local commercial potato production protocols. No chemical growth regulators were applied. For each cultivar, three biological replicates were collected, with each replicate consisting of pooled tuber tissue from five individual plants to reduce within-plot variation. Flesh tuber (with periderm and cortex) were harvested at full maturity (90 days after planting, skin fully set). Each biological replicate comprised 4 g flesh tissue, immediately frozen in liquid nitrogen, and divided into three aliquots. All samples were stored at −80 °C for further use.

### 3.2. Metabolite Extraction and LC-MS/MS for Untargeted Metabolomic Analysis

Frozen samples were ground into powder and mixed with 1000 μL of extraction solution (methanol/acetonitrile/water, 2:2:1, *v*/*v*). The mixture was vortexed vigorously for 30 s, followed by ultrasonication in an ice-water bath (4 °C) for 10 min. To precipitate proteins, the samples were incubated at −40 °C for 1 h. Subsequently, 500 μL of the supernatant was transferred to the wells of a protein precipitation plate. The plate was placed on a vacuum manifold, and vacuum filtration was performed at 6 psi for 120 s. Quality control (QC) samples were prepared by pooling equal aliquots of the supernatant from all individual samples.

LC-MS/MS analyses were conducted on an ultra-high-performance liquid chromatography (UHPLC) system (Vanquish, Thermo Fisher Scientific, Waltham, MA, USA) coupled with a Phenomenex Kinetex C18 column (2.1 mm × 100 mm, 2.6 μm) and an Orbitrap Exploris 120 mass spectrometer (Thermo Fisher Scientific, Waltham, MA, USA) [25]. The mobile phase consisted of phase A (0.01% acetic acid in water) and phase B (isopropanol/acetonitrile, 1:1, *v*/*v*). The column temperature was maintained at 25 °C, and the autosampler temperature was set at 4 °C with an injection volume of 2 μL. The mass spectrometer was operated in information-dependent acquisition (IDA) mode under the control of Xcalibur software (versions 4.1) (Thermo Fisher Scientific, Waltham, MA, USA). The electrospray ionization (ESI) source parameters were optimized as follows: sheath gas flow rate = 50 Arb, auxiliary gas flow rate = 15 Arb, sweep gas flow rate = 1 Arb, capillary temperature = 320 °C, vaporizer temperature = 350 °C, full MS resolution = 60,000, MS/MS resolution = 15,000, stepped normalized collision energy (SNCE) = 20/30/40, and spray voltage = 3.8 kV (positive ion mode) or −3.4 kV (negative ion mode).

### 3.3. Untargeted Metabolomic Data Analysis

Raw MS data were converted to mzXML format using ProteoWizard software (version:3.0.21229). Subsequent feature detection, extraction, alignment, and integration were performed using an in-house R program based on the XCMS package. The final dataset, containing feature IDs, sample names, and normalized feature areas, was imported into SIMCA 18.0.1 software (Sartorius Stedim Data Analytics AB, Umeå, Sweden) for multivariate statistical analysis. Prior to analysis, the data were subjected to autoscaling and logarithmic transformation to reduce the effects of noise and high variable variance. Samples outside the 95% confidence interval in the PCA score plot were identified as potential outliers and excluded from further analysis.

To enhance group separation and screen for differentially accumulated metabolites (DAMs), supervised orthogonal projections to latent structures-discriminant analysis (OPLS-DA) was performed. A 7-fold cross-validation was used to calculate the model parameters R^2^ (explained variance) and Q^2^ (predictive ability). The robustness of the OPLS-DA model was further validated by 200 permutation tests. A lower Q^2^ intercept value indicates a lower risk of overfitting and higher model reliability. Metabolites with variable importance in the projection (VIP) > 1 (from the first principal component of OPLS-DA), *p* < 0.05 (Student’s *t*-test) and |log2(FoldChange)| > 1.0 were defined as DAMs. Finally, pathway enrichment analysis of DAMs was carried out using the Kyoto Encyclopedia of Genes and Genomes (KEGG, http://www.genome.jp/kegg/, accessed on 13 August 2025) and MetaboAnalyst (http://www.metaboanalyst.ca/, accessed on 13 August 2025) databases [26].

### 3.4. RNA-Seq and Analysis

The samples were ground into powder under liquid nitrogen conditions, and the total RNA was extracted using the EZNA Plant RNA Kit (Omega, Shanghai, China). Transcriptome library construction was performed using the Hieff NGS^®^ Ultima Dual-mode RNA Library Prep Kit (Premixed version) (Yeasen, Shanghai, China). Briefly, the target RNA was first subjected to enrichment and fragmentation. Subsequently, the first-strand cDNA was synthesized, followed by the synthesis of the second-strand cDNA. After end repair and dA-tailing, specific MGI^®^ adapters were ligated to the cDNA fragments. The ligation products were then purified and subjected to PCR amplification to obtain the final cDNA library. The RNA libraries were sequenced on the DNBSEQ-T7 sequencing platform by Smartgenomics Technology Institute (Tianjin, China).

The raw sequencing data were filtered and trimmed using SeqPrep (https://github.com/jstjohn/SeqPrep, accessed on 1 August 2025) and sickle (https://github.com/najoshi/sickle, accessed on 1 August 2025). Then the clean data were aligned to the potato reference genome (SolTub_3.0, https://www.ncbi.nlm.nih.gov/datasets/genome/GCF_000226075.1/, accessed on 1 August 2025) using HISAT2 software (version:2.2.1). StringTie software (version:3.0.3) were used to assemble the transcripts for each sample, and then merge the transcripts from all samples into a single transcriptome. The featureCounts tool was used to filter out reads with a mapping quality score lower than 10, unpaired mapped reads, and reads mapped to multiple genomic regions, respectively. The count of the mapped reads from each sample was normalized to fragments per kilobase of transcript length per million mapped reads (FPKM) for each predicted transcript using Cufflinks (v2.2.1). Differentially expressed genes (DEGs) were identified with the criteria of |log2(FoldChange)| > 1.0 & padj <= 0.05. The clusterProfiler software (Version:4.19.6) was used to perform GO and KEGG functional enrichment analyses on the DEGs, with a padj value of less than 0.05 set as the threshold for statistically significant enrichment of pathways and items [27].

### 3.5. Correlation Analysis

The gene-to-metabolite correlation network was constructed using Pearson correlation coefficients calculated between normalized FPKM values of structural genes and relative metabolite abundances (log2-transformed). Only correlations with |r| > 0.80 and *p* < 0.001 (Benjamini-Hochberg FDR corrected) were retained for network visualization. The network was visualized using Cytoscape v3.9.1 with force-directed layout.

### 3.6. RT-qPCR

RNA used for transcriptome was used for RT-qPCR. cDNA was synthesized using the FastQuant RT Kit (Tiangen, Beijing, China). Each gene was analyzed in three biological replicates and three technical replicates. Primers (F: ATTGGAAACGGATATGCTCCA and R: TCCTTACCTGAACGCCTGTCA) were used to normalize gene expression data (https://ngdc.cncb.ac.cn/icg/species/accession/ICG00103, accessed on 1 September 2025). Fold change was calculated using the 2^−ΔΔCt^ method [28]. Details regarding the RT-qPCR primers are listed in Table S10.

## 4. Conclusions

This study, we characterized the metabolic and transcriptional profiles of three potato cultivars with distinct coloration via an integrated metabolomic and transcriptomic approach. Metabolomic analysis identified a total of 1128 metabolites, and revealed that pigmented potato cultivars exhibited a significant accumulation of flavonoids and linoleic acid derivatives relative to the conventional white cultivar. Transcriptomic profiling further detected abundant DEGs among the three cultivars, with notable up-regulation of DEGs in pigmented cultivars in the biosynthetic pathways of terpenoids, flavonoids and linoleic acid, as well as in starch and sucrose metabolism. Integrated multi-omics analysis revealed that the high expression of structural genes in the flavonoid biosynthesis pathway is strongly associated with flavonoid accumulation in pigmented potatoes, suggesting that transcriptional upregulation of these genes may be a key driver of flavonoid biosynthesis. Additionally, several transcription factors from the MYB and WD40 families were identified as potential regulators of flavonoid and anthocyanin biosynthesis in potato, and their functions require further experimental validation. Collectively, our findings establish a foundation for future functional studies and provide candidate genetic factors for marker-assisted breeding of nutritionally enhanced potato cultivars.

## Data Availability

Publicly available datasets were analyzed in this study. The raw RNA-seq data (Accession no. PRJNA1420170) were uploaded to NCBI.

## Supplementary Materials

The following supporting information can be downloaded at: https://www.mdpi.com/article/10.3390/ijms27062881/s1.
